# A kinase-dead natural polymorphism in the canine *Tnni3k* gene

**DOI:** 10.17912/micropub.biology.001164

**Published:** 2024-05-16

**Authors:** Baylee C Westbury, Hirofumi Watanabe, Henry M Sucov

**Affiliations:** 1 Dept. of Regenerative Medicine and Cell Biology, Medical University of South Carolina; 2 Dept. of Pediatrics and Child Health, Nihon Univeristy School of Medicine

## Abstract

Most mammalian cardiomyocytes become polyploid in the neonatal period, concurrent with their loss of proliferative capacity. In mice, natural or engineered mutation of the cardiomyocyte-specific kinase gene *Tnni3k* causes a higher level of diploid CMs and a higher capacity to support proliferation after adult injury. Here, we identified a polymorphism in the canine *Tnni3k* gene that is particularly common in the West Highland White Terrier breed, and show that this variant eliminates Tnni3k kinase activity. Thus, in several species, natural Tnni3k polymorphisms exist that are predicted to contribute to variation in diploid CM level and heart regenerative ability.

**Figure 1. In vitro kinase assay:**
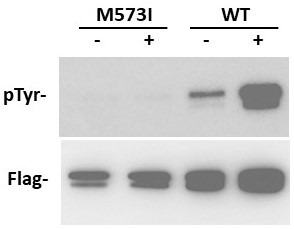
Tnni3k wild-type (WT) and M573I expression plasmids have an N-terminal FLAG epitope that was used for immunoprecipitation. Tnni3k was isolated from transfected HEK293 cell lysates and incubated without (-) or with (+) ATP. Without ATP shows the basal level of Tnni3k phosphorylation as isolated from cells (low because of cellular phosphatases); with ATP shows the autophosphorylation activity of the purified protein. After incubation, Western blotting was performed by probing for phospho-Tyr (pTyr). A parallel blot for FLAG detection shows similar loading.

## Description

Fundamental changes in ventricular cardiomyocyte (CM) biology occur as the mammalian heart transitions from fetal to postnatal life. Two that are relevant to this report are the degree of polyploidy and the ability to proliferate. Proliferation and polyploidy both begin with cell cycle entry and S-phase DNA replication, followed either by successful karyokinesis and cytokinesis to generate new daughter cells (proliferation) or by interruption of karyokinesis or cytokinesis to leave a single cell with double the DNA content (polyploidy). In mice, fetal and early neonatal CMs are diploid and proliferative, but midway during the first postnatal week, CMs mostly become polyploid, and concurrently mostly become nonproliferative. The inability of postnatal CMs to proliferate and thereby regenerate an adequate number of new CMs following heart injury is thought to be a major contributor to the morbidity and mortality associated with myocardial infarction. These observations imply that the fundamental mechanisms that support fetal CM proliferation change in the postnatal myocardium to induce polyploidy and prevent regeneration (Gan et al., 2020) .

Although the vast majority of mammalian ventricular CMs become polyploid, a low percentage of adult CMs are diploid. Labeling studies during the neonatal period show that adult mouse diploid CMs mostly entered cell cycle and completed it to remain diploid, rather than persisting from fetal life without cell cycle activity (Watanabe et al., 2023) . Because there is so little cardiomyocyte cell cycle activity in the uninjured postnatal heart, a higher degree of adult diploid CM content corresponds to a higher degree of CM cell cycle completion during the early neonatal period. In a past survey, we observed that the percentage of diploid ventricular CMs in the adult mouse varies considerably across 120 inbred strains of mice (Patterson et al., 2017) , indicating that the mechanistic features that influence cell cycle interruption or completion have a strong genetic component. By genome-wide association we identified polymorphic loci in the mouse genome that contribute to variation in adult CM ploidy. One such locus includes the CM-specific kinase gene *Tnni3k* , for which a naturally occurring null allele is homozygous in numerous inbred mouse lines (Wheeler et al., 2009) . We confirmed *Tnni3k* as the relevant gene in this locus by showing that homozygosity of an engineered null allele resulted in a 3-fold higher level of diploid CMs compared to isogenic control mice (Patterson et al., 2017) . The same increase occurred when we introduced a K489R point mutation into mice that creates a kinase-dead but stable Tnni3k protein (Gan et al., 2019) , indicating that kinase activity is required for its inhibitory influence on cell cycle completion. Consistent with the hypothesis that CM ploidy is a surrogate indicator of regenerative ability, we showed that *Tnni3k* mutant mice have enhanced cellular regeneration after adult heart injury (Patterson et al., 2017, Purdy et al., 2023) .

While mice provide the easiest vehicle for demonstrating genetic-based variation in CM ploidy and regeneration, we are also interested in the extent to which these may be variable in other species. We previously showed that a prevalent human *TNNI3K* kinase domain polymorphism (Ile686Thr; allele frequency roughly 2% worldwide) is severely hypomorphic for kinase activity (Gan et al., 2019) . We introduced the corresponding mutation into the mouse germline and showed that this increased diploid CM frequency just as seen with the full null and K489R kinase-dead alleles (Gan et al., 2021) .

Dogs are an additional genetic resource because of the extensive inbreeding that has occurred during the development of individual breeds. In a review of the Dog Genome Project database (Plassais et al., 2019) , we noted in the West Highland White Terrier breed the particularly high frequency of a Met574Ile polymorphism (allele frequency 8/22 = 36%) in the kinase domain of the canine *Tnni3k* gene. This variant was also found in individuals of a few other breeds; the equivalent mutation (rs199899961) is also found at a very low frequency in the gnomAD database of human polymorphisms.

Here, we tested the functional consequences of the canine M574I product by introducing the corresponding mutation into the framework of the mouse *Tnni3k* cDNA in an N-terminal epitope-tagged expression construct. Following procedures that we have previously used to determine the kinase activity of numerous Tnni3k variants (Gan et al., 2019) , HEK293 cells were transfected with expression constructs and cell lysates prepared. Tnni3k protein was isolated by immunoprecipitation directed against the epitope tag, and the kinase activity of the isolated protein determined in an in vitro autophosphorylation assay. Tnni3k is a mixed Ser/Thr and Tyr kinase, and here as in the past (Gan et al., 2019) we used an anti-phospho-Tyr antibody to visualize the degree of autophosphorylation. In this assay, after immunoprecipitation but in the absence of added ATP there is only marginal phospho-Tyr signal, which reflects the low phosphorylation state of the protein as it is isolated from the transfected cells (presumably because of the activity of cellular phosphatases). In the presence of ATP, the purified wild-type protein demonstrates robust kinase (autophosphorylation) activity. The M573I mutant protein (now using the mouse protein numbering, which is one less than for dog), however, had negligible kinase activity (see the Figure). Parallel blots visualized for the epitope tag confirmed the presence of immunoprecipitated protein in these assays. We conclude that the canine M574I variant is kinase-dead.

A caveat of the approach is that this is a biochemical assay using protein artificially expressed in a non-cardiomyocyte cell type, such that results do not necessarily reflect the activity of the kinase in its natural setting.

Mice that are homozygous for the natural *Tnni3k* null allele, the engineered *Tnni3k* null allele, and the K489R kinase-dead allele all appear to be healthy and live a normal lifespan. Similarly, although millions of people are homozygous for the human I686T hypomorphic allele, this is not known to be associated with any clinical consequence. The West Highland White Terrier breed is likewise not known to be particularly susceptible to any heart-related issues (Fleming et al., 2011) . Because *Tnni3k* mutations have no overt phenotype in mice or humans, it is unlikely that the canine M574I variant was the basis of its selection during derivation of this breed. Rather, we suspect that the high allele frequency of this variant in this breed represents its coincidental presence in the small number of dogs from which the breed was originally derived (i.e., a founder effect).

Despite there being no obvious consequence from *Tnni3k* mutations, subtle alterations, for example in the percentage of diploid CMs in the heart or differences in regeneration after adult heart injury, are not evident without experimental measurement, which cannot be done in humans and would not be appropriately done in dogs either. Nonetheless, the conceptual principle of our mouse studies, that naturally occurring genetic polymorphisms cause variation in the diploid/polyploid CM ratio across a population, could be extended to humans from our prior work and can now also be extended to dogs. Because this ratio reflects the propensity of CM cell cycle completion vs. interruption, it is reasonable that different people and different dog breeds will have different degrees of CM regeneration after infarction, just as experimentally demonstrated for different inbred mouse strains (Patterson et al., 2017, Salimova et al., 2019) . In other words, heart regeneration need not be uniformly low in all mammalian species or in all individuals of a given species. *Tnni3k* is one gene that is naturally polymorphic in multiple species that is likely to influence variation in this outcome.

## Methods

Dog genome data. Variant calls relative to CanFam3.1 were downloaded as a VCF file from https://research.nhgri.nih.gov/dog_genome/index.shtml , and polymorphisms in the canine *Tnni3k* gene sorted by breed and position. Of 11 West Highland White Terrier individuals in this database, the M574I variant was heterozygous in 6 and homozygous in 1.

Plasmid construction. The full-length wild-type mouse *Tnni3k* open reading frame, modified to include an N-terminal FLAG epitope, was cloned into pcDNA3.1. The M573I variant (following the mouse numbering) was made from this template by oligo-directed PCR mutagenesis and the resulting plasmid was sequenced in its entirety to confirm the absence of unexpected mutations. Two separate plasmid preparations were used independently with identical results.

Cell culture and protein purification. Human embryonic kidney (HEK293) cells were cultured in DMEM with 10% FBS, 1% Pen-Strep, 1% nonessential amino acids, 1% sodium pyruvate, and 4.5 g/l L-glutamine. Cells in 6 cm dishes at 80% confluence were transfected with 500 ng of purified plasmid using Invitrogen Lipofectamine 3000 Transfection Kit (cat# L3000-008) and Gibco Opti-mem (cat#31985-062). Media was replaced after 24 hours and lysates were collected after 48 hours in lysis buffer (1% NP40, 50mM Tris pH = 7.4, 150mM NaCl). Lysates were then placed on a rocker at 4°C for 2 hr and the supernatant collected after centrifugation at 16,000rpm for 20 min.

For the in vitro kinase assay, 200 µg of transfected HEK293 cell lysate was incubated with 2 µl of anti-Flag antibody (Sigma #F1804) overnight with agitation at 4°C. Magnetic Dynabeads (Invitrogen #10003D) were incubated with the protein and antibody mixture for 2 hr at 4°C with agitation. DynaMag-2 (Invitrogen #12321D) was used to separate the beads from the suspension. The beads were washed 3 times with agitation in TBST and resuspended in 10 µl lysis buffer then incubated with or without ATP at 30°C for 30 min with agitation. After incubation, protein was eluted from the beads using 50 mM glycine (pH = 2.3) to which was added ¼ volume of 5x loading buffer (5x = 10% bME, 50% glycerol, 0.1M Tris pH=6.8, 10% SDS) and the samples heated at 70°C for 10 min. Western blotting was performed using anti-phospho-Tyr antibody (Cell Signaling #9411S) and anti-FLAG antibody (Sigma #F1804).
